# Professional decision-making in medicine: Development of a new measure and preliminary evidence of validity

**DOI:** 10.1371/journal.pone.0228450

**Published:** 2020-02-07

**Authors:** Alison L. Antes, Kelly K. Dineen, Erin Bakanas, Tyler Zahrli, Jason D. Keune, Matthew J. Schuelke, James M. DuBois

**Affiliations:** 1 Division of General Medical Sciences, Department of Medicine, Washington University School of Medicine, St. Louis, Missouri, United States of America; 2 Creighton University School of Law, Omaha, Nebraska, United States of America; 3 Division of Palliative Medicine, Department of Medicine, Washington University School of Medicine, St. Louis, Missouri, United States of America; 4 Saint Louis University School of Medicine, St. Louis, Missouri, United States of America; 5 Departments of Surgery and Health Care Ethics, Saint Louis University, St. Louis, Missouri, United States of America; 6 Research Strategy Group, Saint Louis University Office of the Vice President for Research, St. Louis, Missouri, United States of America; Chinese Academy of Medical Sciences and Peking Union Medical College, CHINA

## Abstract

**Introduction:**

This study developed a new Professional Decision-Making in Medicine Measure that assesses the use of effective decision-making strategies: seek help, manage emotions, recognize consequences and rules, and test assumptions and motives. The aim was to develop a content valid measure and obtain initial evidence for construct validity so that the measure could be used in future research or educational assessment.

**Methods:**

Clinical scenario-based items were developed based on a review of the literature and interviews with physicians. For each item, respondents are tasked with selecting two responses (out of six plausible options) that they would choose in that situation. Three of the six options reflect a decision-making strategy; these responses are scored as correct. Data were collected from a sample of 318 fourth-year medical students in the United States. They completed a 16-item version of the measure (Form A) and measures of social desirability, moral disengagement, and professionalism attitudes. Professionalism ratings from clerkships were also obtained. A sub-group (n = 63) completed a second 16-item measure (Form B) to pilot test the instrument, as two test forms are useful for pre-posttest designs.

**Results:**

Scores on the new measure indicated that, on average, participants answered 75% of items correctly. Evidence for construct validity included the lack of correlation between scores on the measure and socially desirable responding, negative correlation with moral disengagement, and modest to low correlations with professionalism attitudes. A positive correlation was observed with a clerkship rating focused on professionalism in peer interactions.

**Conclusions:**

These findings demonstrate modest proficiency in the use of decision-making strategies among fourth-year medical students. Additional research using the Professional Decision-Making Measure should explore scores among physicians in various career stages, and the causes and correlates of scores. Educators could utilize the measure to assess courses that teach decision-making strategies.

## Introduction

Physician professionalism supports interactions among health care professionals, promotes patient care, and preserves public trust in medicine [1–3]. Professionalism is considered an essential competency of physicians and is of major interest to medical educators [4–6]. Inculcating professionalism among medical students and trainees involves formal and informal learning in the classroom and in clinical settings [7, 8]. When physicians practice medicine, no matter their career stage, professionalism requires an intentional approach to navigating the duties of a physician responsibly [9, 10]. However, professionalism is a complex, multifaceted phenomenon that is difficult to define and measure, making it a challenge for researchers to study and for educators to teach and assess [11–17].

In the current study, we report on the development and content validity of the Professional Decision-Making in Medicine Measure (PDM), along with an initial study of the PDM’s construct validity using a sample of fourth-year medical students in the United States (U.S.). The PDM assesses decision-making strategies professionals can employ to choose appropriate responses in challenging situations [18]. This approach recognizes that professionalism is a complex interplay of individual and context; thus professionals require “meta-skills” that foster ethical, effective decisions and actions [4]. That is, effective professional decision-making for physicians, whether students, residents, or physicians well established in their careers, requires complex judgments. For instance, these judgments must manage competing interests, account for what is clinically appropriate, consider patient values, and address conflicts between patients, families, and medical professionals [10, 19–22].

In these situations, ethical principles (e.g., justice and beneficence) and professional standards (e.g., honesty and respect) provide some guidance, but the physician must recognize the dynamics of the issue, address subjective emotions, examine underlying assumptions, and weigh potential consequences to make the best professional decision [18]. For example, consider the following situation:

A patient is hospitalized and being treated with intravenous antibiotics for a third post-operative bacterial infection. The patient requests to be sent home with oral antibiotics. The physician is concerned that discharge is not in the patient’s best interest. However, the medical director just called and indicated the patient no longer meets criteria to continue hospitalization and should be discharged.

The physician’s decision in this situation is not straightforward. Handling complex professional challenges requires reasoning strategies that compensate for novelty, complexity, and uncertainty [18, 23, 24].

A set of five decision-making strategies—seek help, manage emotions, anticipate consequences, recognize rules and context, and test assumptions and motives—can assist professionals with navigating complicated professional decisions. They can offset self-serving and faulty decisions due to bias, negative emotions, stress, or lack of experience or information [10, 25, 26]. Bias is particularly important to compensate for because it is implicated in physician decision-making that perpetuates health disparities and inappropriate influences of conflicts of interests [10, 27–29]. Effective engagement of these strategies also assists with assessing the interpersonal context and dynamics of a particular situation, which can help a professional gather relevant information and communicate decisions in a manner that controls emotions and recognizes the needs of others [30]. Moreover, self-reflection, which is important for professional self-monitoring and ongoing growth, is encouraged by routinely employing these strategies [10, 31]. Table 1 summarizes the strategies and key compensatory mechanisms identified in psychological research and operationalized in the PDM response options [18]. The strategies are conceptually overlapping and may be engaged in any order based on the nature of a particular situation.

**Table 1:** Decision-making strategies operationalized in the PDM response options.

	Description	Strategy compensates for
**S****eek Help**	Ask for advice from an objective individual; seek institutional resources; consider what others have done in similar situations	*Lack of information*, *or incomplete information* Lack of requisite knowledge or experience Hasty, biased, or emotional judgment and decision-making Incomplete appraisal of the situation, personal motives, or alternatives
**M****anage Emotions**	Assess and regulate emotional responses to the problem that can hinder objectivity, judgment, and interactions with others	*Hasty*, *biased*, *or emotional judgment and decision-making* Competing interests and demands Biased or incomplete appraisal of the situation, personal motives, or alternatives
**A****nticipate Consequences**	Consider possible outcomes, including the likely short- and long-term consequences of potential decision alternatives; recognize others’ perceptions, concerns, and motivations; predict the likely effects of alternatives on oneself and others	*Incomplete integration of facts*, *cues*, *alternatives*, *and potential outcomes* *Biased or shortsighted thinking about alternatives*, *or failure to consider creative alternatives* Hasty, biased, or emotional judgment and decision-making Incomplete appraisal of the situation, personal motives, or alternatives
**R****ecognize Rules and Context**	Be aware of relevant rules, guidelines, and principles that should apply to the situation; recognize individuals involved and the dynamics between them; identify key goals and causes in the situation	*Incomplete analysis of situational causes*, *norms*, *and rules* *Biased assessment of the roles*, *perspectives*, *needs*, *or motivations of others* Hasty, biased, or emotional judgment and decision-making
**T****est assumptions and motives**	Scrutinize interpretation of the problem, situation, and potential alternatives; evaluate personal motivations, values, and goals and how they might affect judgment and decision-making	*Hasty*, *biased*, *or emotional judgment and decision-making* *Incomplete or biased analysis of situational causes*, *norms*, *rules*, *alternatives*, *outcomes*, *or faulty interpretation of roles*, *perspectives*, *needs*, *or motivations of oneself or others* Lack of requisite knowledge or experience Lack of information, or incomplete information

Adapted from DuBois et al. 2016. Italics indicate the primary compensatory mechanisms; the remaining statements represent secondary mechanisms. In the PDM response options, anticipate consequences and recognize rules/context are reflected in the same options, as anticipating consequences necessarily involves recognizing rules/context, and recognizing rules/context is a facet of anticipating consequences. Thus, PDM response options are scored according to 4 strategies: seek help, manage emotions, recognize consequences and rules/context, test assumptions. As illustrated with the underlined letters, the mnemonic “SMART” provides a useful tool for remembering the strategies.

Delineating professionalism as relying, at least in part, on decision-making strategies has two key advantages in its implications for educational approaches. First, it is not feasible to inform students and professionals of all potential challenges they might encounter in their careers. In addition to learning key principles and standards, which are essential but incomplete for making effective professional decisions, professionals need to learn strategies for examining situations and potential choices, while considering personal emotions and assumptions. Thus, inculcating decision-making strategies supports professionalism across complicated clinical situations. Second, this practical approach aligns with values-based professionalism. The strategies promote reflecting on personal values to enact virtues, and they encourage applying professional rules and principles to challenges [2, 4, 10].

Educational programs that adopt a decision strategies approach are appropriate for physicians at all career stages and could be delivered in variable formats. For example, the strategies could be incorporated into medical student courses focused on medical professionalism and ethics, particularly through application to cases. Medical students could also be prompted to apply the strategies in reflection on real-world experiences in their clinical rotations. The strategies could be taught in shorter training programs aimed at professional development or continuing education for more advanced physicians; here inviting application of the strategies to personal cases is particularly instructive. Strategies could be taught during postgraduate education by prompting residents to journal on experiences in the clinical setting and apply the decision strategies. This reflection could be followed by a facilitated peer discussion. All of these instructional activities ultimately aim to cultivate application of the strategies in the workplace. Educational interventions focused on decision strategies have been effective for researchers at various career stages [32, 33]. In these training programs, teaching the strategies includes providing learners with a mnemonic, “SMART,” for remembering the five strategies (see the note in Table 1).

Any educational interventions that explicitly teach these decision strategies, or aim to develop skills like situational appraisal, bias management, self-reflection, and emotion management [30, 34, 35], should yield pre-post changes in strategies measured by the PDM. Ideally, the PDM would be administrated in a pre-post fashion where pre assessment takes place before education, and the post measurement occurs following the educational intervention. Follow-up measurement occurring in the months after the educational program would offer evidence of long-term retention of the strategies.

## Methods

This study developed a new measure of decision-making strategies not previously reported on elsewhere. We also obtained initial data on the measure to establish its usefulness for future research on decision-making or for use in educational assessment. First, we describe the development of the measure which focused on its content validity. Then, we describe obtaining evidence of the measure’s construct validity in a sample of medical students in the U.S.

### Development of the PDM instrument

The scenario-based measurement approach presents vignettes and asks participants to select responses. This approach aims to simulate the kind of decision-making that would be necessary in that professional situation [36]. The rationale for measuring decision-making strategies specifically is three-fold: strategies can be learned in educational interventions, strategies are applicable to any medical specialty or clinical setting, and the empirical literature on improving decision-making strongly supports the strategies [18]. Additionally, the new measure of decision strategies asks participants to select their responses according to what they *might do* if in the situation to examine behavioral intentions, rather than just cognition.

We designed the PDM instrument in a similar fashion as a measure of Professional Decision-Making in Research (PDR) that has demonstrated validity and reliability among over 900 researchers in varied career stages [18, 37]. Our process included identifying the content to be covered by the measure, substantiating the comprehensiveness of the identified content and collecting example professional challenges by speaking to practicing physicians, writing and revising the scenarios and items as a team, and, finally, conducting cognitive interviews with practicing physicians to ensure the scenarios, items, and response options were clear and plausible. First, our efforts focused on designing a 16-item measure (Form A); then we adapted Form A to create a second, alternate measure, Form B. We sought to develop two forms so that the measure could be used for pre-posttest assessment. After describing the development of Form A, we describe the adaption of Form A to create Form B.

#### Establishing the content to be addressed in the PDM

To ensure content validity, we developed items representative of the multifaceted nature of professionalism in medicine. We identified topics from the American Board of Internal Medicine’s physician charter [9] and the Accreditation Council for Graduate Medical Education’s core competencies [38]. Next we compared those topics to a practical clinical ethics reference book [39] and key professionalism frameworks and reviews [14, 15, 17, 40–44]. This process yielded 14 potential domains of professionalism, and the specific issues within the broader domains, to address in PDM items (see Table 2). The domains were identified through a content analysis [45] performed by AA and TZ of the reference materials and existing frameworks, and they were reviewed by the remaining team members.

**Table 2:** Professionalism content domains covered by PDM items.

Broad Domain	Description of Specific Issues
Confidentiality	Protect confidential patient information (within legal constraints)
	Honor patient privacy
Interpersonal Communication	Facilitate dialogue with patients
	Maintain honesty in communication
Clinical Competence	Understand policies and procedures at one’s place of practice
	Maintain clinical knowledge and competence
Conflicts of Interest	Manage potential conflicts associated with industry collaboration
	Follow professional standards pertaining to acceptance of gifts
	Allocate medical resources according to patient best interest
Improving Quality of Care	Adhere to standard of care; reduce medical error, optimize outcomes, and increase patient safety
	Encourage quality assessment to strive toward continuous improvement in care
Improving Access to Care	Reduce barriers to health care through individual and collective means
	Advocate for promotion of preventive medicine and public health measures
Collaboration	Integrate inter-professional health care disciplines when clinically desirable
	Seek consult and provide referrals when patient’s care needs are beyond the scope of one’s practice
Accountability and Oversight	Prevent overuse and abuse of medical resources
	Respond to mistakes (made personally or by colleagues)
	Address inappropriate, risky, or illegal behavior
Business Ethics and Legal Compliance	Billing, insurance, fraud, lying about services, reimbursement
	Legal issues in the business of medicine
Relationships with Patients and Families	Demonstrate compassion for patients and sensitivity to patient needs and cultures
	Interact with patients and families respectfully and transparently
	Foster trust in the physician-patient relationship
Relationships with Colleagues	Interact with physicians, nurses, medical students, and residents respectfully
	Avoid abuses of power, harassment issues, and verbal disrespect
	Maintain collegiality and fairness in the work setting
Withholding and Withdrawing Futile Treatments	Know and follow institutional policies
	Acknowledge the diversity of patient goals for treatment
	Follow best practices (e.g., time limited trials, calling ethics consults)
Distinguishing between Innovative Medicine and Research	Know when a procedure requires IRB or peer review
	Recognize when prescribing off-label is appropriate
Obtaining Voluntary Informed Consent	Convey medical information in an understandable manner
	Assess decisional capacity and recognize appropriate decision-makers
	Avoid coercion or undue influence

Domains were identified through review of key guideline documents, a medical ethics reference manual, and professionalism literature, and they were confirmed through informant interviews.

To further support the content and face validity of items, we conducted one-hour semi-structured interviews with 12 physicians from varied specialties in academic and community practices. Informants included five internists, two neurosurgeons, two pediatricians, one nephrologist, one geriatrician/endocrinologist, and one general surgeon. We obtained real-world physician experiences to inform item generation and confirm the 14 content domains. AA and TZ conducted the interviews in person at the physicians’ offices. Our interview guide asked, “What professional challenges or dilemmas have you wrestled with as a physician? Can you tell us about these challenges?” We directed informants to consider the full range of challenges, responsibilities, and interactions in their work and used follow-up prompts for additional details when necessary. Interviews were audio recorded and transcribed.

Themes in the transcripts were identified by AA and TZ using thematic analysis [46]. They reviewed each transcript and generated short conceptual codes reflecting the key issues within the narrative (e.g., “relationship with patient” or “conflict with colleagues”) and continued generating codes until no new codes emerged. They marked passages of text with the applicable code(s) in two stages: codes were applied first during the code generation process, and then again in a second pass through the transcripts to ensure all applicable codes that emerged had been applied. Next, AA and TZ mapped each excerpt of coded narrative to the 14 domains identified in the literature review. Most stories were coded with themes that related to multiple of the 14 domains, and importantly, all of the themes fit within the 14-domain framework, endorsing its comprehensiveness.

#### Writing PDM scenarios, items, and response options

The item writing team has expertise in medicine, health law, health care ethics, psychology, and measure development and validation. We sought to develop items accessible to medical students, residents, or practicing physicians with a general medical education so the measure would be relevant in different contexts. JD, KD, and EB drafted initial scenarios, items, and response options using the stories from informant interviews as ideas for potential scenarios and vignette items. The PDM addresses complicated situations that are realistic to the challenges encountered by physicians. For instance, making a choice might require handling competing concerns, such as what is in the best interest of a patient versus how a decision might impact one’s standing at the institution. After initial scenarios, items, and responses were drafted, all authors reviewed them for clarity and readability, along with medical accuracy and plausibility. We held three team meetings to review, discuss, and revise items.

Table 3 presents an example PDM item to illustrate the structure of the instrument. Notation labels the overarching scenario, vignette item, and response options. The measure consists of five overarching scenarios that provide context for the three to four vignette items following each scenario (16 vignette items total). These vignette items present the professional problem encountered by the physician which operationalizes the professionalism domains in Table 2. Each item includes 6 response options. Three represent a decision-making strategy, which operationalizes the strategies described in Table 1, and are scored as correct responses. These options reflect effective first-line decisions in the situations. The remaining three response options represent a violation of a strategy and are scored as incorrect, although these response options are plausible. These responses are often premature choices or could lead to problems if selected in a real-world situation. The instructions indicate: “Select the two options that best describe what you might do if you were really in the challenging situation.”

**Table 3:** Sample PDM item.

*(Overarching Scenario)*
You are an internist in a large privately-owned physician practice group in an area with a high percentage of older Medicare patients. You have worked for the practice for 5 years. You see a lot of medically complex patients.
*(Vignette Item)*
You are seeing a 64-year-old female patient who has been under your care for several years. She has come for a follow up visit to discuss her recent abnormal mammogram. She has brought one of her adult daughters with her. The radiologist has recommended a biopsy, and you feel your patient is healthy enough to proceed with this testing. You arrange an appointment for the biopsy. The next day the patient’s daughter calls. She asks that you not share the biopsy results with the patient, but that you inform her first so she can decide what to do with the information. Consider the following options:
*(Response Options with decision-making strategy scoring)*
a. Ask the daughter if her siblings agree with her request. *(strategy not employed*, *incorrect response)*
b. See if the patient has appointed a health care power of attorney. *(strategy not employed*, *incorrect response)*
c. Ask your patient if she wants her results or prefers you share them with her daughter. *(strategy employed*, *recognize consequences and rules*, *correct response)*
d. Tell the daughter you cannot comply with her request. *(strategy not employed*, *incorrect response)*
e. Ask one of your senior colleagues what he or she would do. *(strategy employed*, *seek help*, *correct response)*
f. Ask the daughter to tell you more about her concerns. *(strategy employed*, *test assumptions*, *correct response)*

Italics are notations for the reader; the participant version did not include this notation.

After we finalized our draft measure, we conducted cognitive interviews with six experienced physicians in academic medicine [47]. The aim was to obtain their feedback regarding the clarity of the scenarios and vignette items, and the relevance and plausibility of the response options. We also inquired about which response options they viewed as most appropriate to identify if our scoring of the responses as correct or incorrect aligned with their assessment. This process resulted in minor edits to the scenarios, items, and response options; the edits primarily focused on clarifying or simplifying wording. Before using the items in our validity study, we examined the Lexile scores of the scenarios, items, and response options to ensure readability and increase the validity of responses from participants who speak English as a second language. The Lexile scores were, on average, about 1,000 (approximately the 8^th^ grade level).

#### Creating an alternate PDM form

In year 1 of the project, we developed a 16-item measure, “Form A.” In year 2, we developed an alternate 16-item measure, “Form B,” so it would be feasible for educators to implement the PDM in a pre-post design. The rationale for an alternate form is to prevent posttest responses based on memory of the pretest; and more importantly, to maintain respondent engagement by presenting new scenarios and items. Form B was constructed by adapting Form A. Starting with the existing scenarios and items, we adapted the context and characters within the situations to create new scenarios and items. For example, in Form A, an overarching scenario about “a radiologist working at a large community hospital” became in Form B “an internist in a rural hospital.” Adaptation of the vignette items included, for example, translating a challenging interaction with a patient’s daughter to a patient’s son, and changing the nature of the specific problem to fit with the new context presented in the overarching scenario. The general underlying professional challenges (e.g., patients requesting specific medications, requests from superiors to increase patient loads at potential risk to quality of care, interpersonal conflict with medical staff, and handling mistakes of subordinates) remained similar in the Form B vignette items. We composed new response options to correspond to the new items. We ensured that the alternate form included in the response options the same number of each of the decision-making strategies as presented on Form A. Overall, our approach to designing Form A ensured that the central decision-making tasks would be similar between the two forms and therefore comparable, but that the experience of completing Form B would be distinct from Form A and thus engaging.

### Construct validity study

We investigated the construct validity of the new PDM measure in a sample of fourth-year medical students in the U.S. In addition to the PDM, they completed three additional measures (i.e., social desirability, moral disengagement, and professionalism attitudes). We also obtained ratings of the students’ professionalism from their clinical clerkship rotations. Our aim was to provide initial construct validity evidence by examining the correlations of PDM scores with these additional measures. Specifically, we aimed to show convergent validity in PDM scores being related to moral disengagement, and discriminant validity in PDM scores not being related to social desirability or professionalism attitudes. Evidence for criterion-related validity would be demonstrated by associations of PDM scores with professionalism behavior in the clinical clerkships.

#### Participants

We obtained a convenience sample of medical students (N = 318) in the final month of medical school at Saint Louis University. We collected data from two medical student cohorts over two years (year 1, n = 152; year 2, n = 166) to accrue enough data to obtain stable relationships and ensure variation in PDM scores. We estimated at least a modest correlation (r = -.20) of moral disengagement and professional decision-making based on the moderate effect size among faculty researchers (r = -.31) [37]. A power analysis using a correlation of .20, alpha of .05, and power of .80, projected a necessary sample size of 194. Thus, we collected data in two medical student cohorts to obtain a sample size that would be more than adequate.

All participants completed Form A of the PDM. In year 2, we pilot tested Form B in a subgroup (n = 63) of the full sample. The full sample was 54% female, and 72% were 26–30 years of age. Additionally, 61% reported their race as White, 32% as Asian, 4% Black, and 3% other; 16% reported being born outside of the U.S. and 11% English as their second language. There were minimal demographic differences between those who did and did not volunteer to pilot test Form B. The Form B sample was 59% female, 67% 26–30 years of age, and 67% White.

#### Ethical review

The Saint Louis University Institutional Review Board provided ethical review for the study (IRB#26948). They approved the study under expedited review and approved a waiver of written consent. At a scheduled session of a required class, a member of the research team provided the potential participants with a written statement describing the opportunity to participate in a 20–30 minute, voluntary research study (the statement was also read aloud). Participants returned a form to the investigator indicating their preference to participate or abstain from the study. Participants were offered gifts cards for participation in this study.

#### Measures and procedure

Participants received a link via email to the study questionnaires administered via Qualtrics and completed the measures while convened for the class session. All of the participants in Years 1 and 2 (N = 318) completed PDM Form A and the three validation measures. A smaller cohort in Year 2 (n = 63) also volunteered to complete the pilot test of Form B via Qualtrics and did so following the class session and a lunch break.

Participants completed the new scenario-based *PDM* that instructed them to imagine encountering professional challenges. Specifically, the instructions read: “This survey is intended to give you a sense of how you make professional decisions. You are asked to imagine yourself in a series of different roles as a physician. For each role, you are presented with a series of challenges. Each challenge is followed by six options. From the list of options provided, select the two options that best describe what you might do if you were really in the challenging situation.”

To score responses, participants received one point indicating a correct response for an item when they selected two response options (out of the three correct and three incorrect options provided) reflecting decision-making strategies. Thus, total PDM scores (on Form A and B) can range from 0 to 16. A second, alternative scoring approach produces “strategy preference profiles.” These scores are useful for learner feedback [18]. The four strategy preference scores are computed as a percentage (number of times a strategy was selected/number of times the strategy was presented as an option). For example, a learner might receive the following percentages for each strategy: seek help (62%); manage emotions (17%); recognize consequences and rules (82%); test assumptions (67%). This profile suggests they tend to particularly select responses that recognize consequences and rules but opt for the manage emotions strategy infrequently. While this individual could still score well on the overall (0 to 16) PDM score, this feedback on strategy selection provides useful insight about their approach to responding.

Participants completed the 13-item *Marlowe-Crowne Social Desirability Scale* that assesses responding in a socially desirable manner [48]. Participants rate the extent to which statements are true or false about their attitudes or behaviors (e.g., “I’m always willing to admit it when I make a mistake”). The measure is scored by adding 1 point for each socially desirable response to the 13 items, with a total possible score of 13 (range 0 to 13). Cronbach’s alpha scale reliability was .73. We expected no association between responses on the PDM and social desirability [18]. This finding would provide evidence that respondents are not simply providing the answers they think are desirable to other people, which is a response bias that compromises the validity of a measure. In particular, a finding of no association of social desirability with the PDM measure counters the potential critique that correct responses are too transparent, so respondents simply select those responses to score well.

Participants completed the 8-item *Propensity to Morally Disengage Scale* [49]. Moral disengagement is distancing oneself from ordinarily embraced ethical standards. Individuals can convince themselves that ethical standards do not apply through cognitive distortions including victim blaming, assuming the worst, euphemistic labeling, and minimizing harms [50]. Therefore, they can behave unethically and view it as acceptable. Participants indicate their agreement (1 –*strongly disagree* to 7 –*strongly agree*) with statements, such as “People who get mistreated have usually done something to bring it on themselves,” that reflect cognitive distortions. The measure is scored by computing the mean of responses to the 8 items. Cronbach’s alpha was .72. We expected negative correlations between moral disengagement scores and PDM scores [37].

The 36-item *Penn State Professionalism Questionnaire* (PSPQ) examines attitudes about medical professionalism. Participants rate how much statements represent important elements of medical professionalism (for example, “meets commitments and obligations in a conscientious manner”). The seven scales are accountability, enrichment, equity, honor and integrity, altruism, duty, and respect, and are computed as the mean of the items comprising each scale [51]. Cronbach’s alphas were .79, .83, .74, .77, .76, .71, .26.^b^, respectively. (The respect factor is comprised of two items, contributing to its low reliability.) Our aim was to provide evidence that the PDM measures a construct distinct from attitudes; thus, we anticipated low to modest correlations of PSPQ scales with PDM scores.

We obtained two indicators of professionalism from the students’ clinical clerkships. Faculty educators reported these ratings of students during their clerkships. The medical student education office provided our research team with these existing data. (It is of note that we considered, but did not obtain, disciplinary actions data because the sample size for such cases was too small to be useful for analysis.) Although professionalism ratings by faculty educators are not without limitations [17, 52], our aim was to examine a potential source of criterion-related validity. Criterion-related validity provides construct validity evidence by showing that a test score is related to an expected outcome (e.g., behavior in the workplace) [53].

We obtained professionalism ratings from two different contexts within the clerkship experience. The first professionalism rating, which we refer to as the *Clinical Clerkships Rating*, was reported by the faculty supervisors of the seven required clerkship rotations. Supervising faculty rated students from 1 to 9 (*outstanding*) according to whether they demonstrated “reliability, initiative, honesty, integrity, and punctuality” in each of the seven clerkship rotations. Different faculty supervisors rated different students within each clerkship, and the total number of faculty ratings for a student in any one clerkship was not consistent. Thus, we averaged all available ratings within a clerkship for each student to calculate a reliability coefficient (alpha = .69). We then composed a professionalism score for each student by calculating an average across clerkships weighted by total observations per clerkship. Clerkship ratings suffered from range restriction (with most students rated high), which attenuates correlations.

The second professionalism rating, which we refer to as the *Peer Interactions Rating*, was based on an assessment of the student’s behavior in small group, case-based discussions during the family medicine clerkship. This assessment provided by the faculty facilitator of the discussion sessions was a single rating of the item “Demonstrates professionalism,” using the scale 1 (*below average*) to 5 (*above average*). Therefore, this rating reflected faculty facilitators’ assessments of professionalism in peer-to-peer interactions within a small group discussion setting, whereas the clinical clerkships rating was an assessment of their behavior in the clinical setting. Like the clerkships rating, the peer interactions ratings were fairly high; however, the peer interactions ratings had greater variance.

The final measure included in the study was a brief *Demographic Questionnaire* that assessed gender, age, race, and birth nation.

#### Data analysis

We focused our analyses of the data (S1 File) on the full sample (N = 318) that completed Form A. The key relationship we examined regarding Form B was its correlation to Form A. We examined descriptive statistics for all of the variables and performed correlational analyses to test the associations between the variables. We used non-parametric Spearman’s rank-order correlations because the PDM and validation measure distributions were skewed. We also produced descriptive statistics for the four strategy preference profiles scores. Data were aggregated in R 3.3.3 (R Foundation for Statistical Computing, Vienna, Austria), and analyses performed in IBM SPSS Statistics for Windows, version 24.0, released 2016 (IBM Corp., Armonk, NY, USA).

## Results of validity study

The mean score on Form A (N = 318) was 12.04 (SD = 2.13). Thus, on average, participants answered 75% of items correctly. The observed score range was 1 to 16, with a median of 12. The distribution of scores was negatively skewed (S1 Fig). There was a tendency towards higher scores and a few quite low scores in the long left tail. This is consistent with findings from the PDR [18, 23, 37]. We expected the PDM to be a “mastery test” in which performance indicates that an individual has or has not attained mastery of a subject [54]. Thus, we anticipated most participants would demonstrate adequate use of strategies. Yet, we observed 33% of participants (n = 105) scoring 11 or below (which is 69% or fewer correct).

We also examined the PDM “strategy preference profiles” that are useful for learner feedback in an educational context. These scores are expressed as the percent of times respondents selected a strategy when it was presented as an option. The Form A mean preference profile scores (in decimal form) were as follows: test assumptions (M = .73, SD = .12), recognize consequences and rules (M = .64, SD = .11), seek help (M = .43, SD = .13), and manage emotions (M = .37, SD = .26). These results indicate that participants tended to opt for responses focused on testing assumptions and recognizing consequences and rules, selecting these 73% and 64% of the times they were offered. However, seeking help and managing emotions options were less preferred; these were selected just 43% and 37% of the times they were presented.

We also examined PDM Form B’s descriptive statistics (n = 63). The mean score was 11.86 (SD = 2.74); the observed range of scores was 2 to 16 and the median 13. Thus, the overall mean was slightly lower and the variance slightly greater on Form B. These differences in performance on the PDM Form A and B were not statistically significant. A Wilcoxon signed-rank test showed no statistically significant difference in the Form A scores (M = 12.13, SD = 2.00, Median = 12, IQR = 3) and the Form B scores (M = 11.86, SD = 2.74, Median = 13, IQR = 3), Z = -.564, p = .573. To examine the potential for bias in those who chose to complete Form B, we compared the Form A scores of those individuals who volunteered to complete Form B to those who did not complete Form B. Form A scores for those participants who volunteered to complete both forms (M = 12.13, SD = 2.00, Median = 12, IQR = 3) versus those who did not (M = 12.02, SD = 2.16, Median = 12, IQR = 2) indicated no statistically significant differences, Mann-Whitney U = 7742.0, p = .652. The parallel form reliability coefficient (the correlation between Form A and B) was .61. This pilot test of Form B suggests that the measures demonstrate adequate similarity. Therefore, it would be appropriate to treat Form A as a pretest and Form B as a posttest and compare scores after an educational intervention.

Table 4 shows the correlation matrix and descriptive statistics for all of the study measures. We focused on Form A completed by the full sample to examine construct validity evidence, but preliminary analysis with Form B revealed similar correlations. First, we examined the correlations of demographic variables with Form A scores. English as a second language (r = -.16, p < .01) and reporting Asian as one’s race (r = -.19, p < .01) were negatively related to PDM scores. Reporting White was positively related (r = .24, p < .001). However, being born in the U.S. versus outside of the U.S. was not associated with PDM scores (r = .06, p = .28). Future studies should examine potential explanations for these associations of PDM scores with demographic variables. We do not think they are tied to language challenges in reading the PDM, as the items are written at an 8^th^ grade reading level, and the participants have successfully progressed in their medical studies in an English language medical program. The finding is generally consistent with what we observed among researchers on a measure of professional decision-making [37]. Variables like acculturation to American culture and discernment between behavioral norms, rules, and ideals in the U.S. research workplace, which are higher among researchers from the U.S., are associated with higher professional decision-making scores [37]. More research is needed; however, we do think that there is a need to make explicit cultural beliefs or norms that might influence professional behavior when educating physicians [55–57].

**Table 4:** Correlations among PDM and validation measures.

	1	2	3	4	5	6	7	8	9	10	11	12
1-PDM (Form A)	1											
2-Social Desirability	.03	1										
3-Moral Disengagement	-.16**	-.31**	1									
4-Accountability	.06	.12*	-.32**	1								
5-Enrichment	-.01	.10	-.29**	.66**	1							
6-Equity	.03	.13*	-.45**	.68**	.55**	1						
7-Honor and Integrity	.11	.06	-.43**	.71**	.59**	.67**	1					
8-Altruism	.07	.16**	-.38**	.61**	.54**	.56**	.54**	1				
9-Duty	.05	.14*	-.37**	.73**	.67**	.65**	.72**	.53**	1			
10-Respect	.02	.18**	-.28**	.47**	.40**	.40**	.39**	.38**	.43**	1		
11-Clinical Clerkships Rating^a^	.08	.05	-.12*	-.08	.01	.06	-.01	.09	-.03	.05	1	
12-Peer Interactions Rating^b^	.13*	.04	-.16**	.12*	.11	.15*	.05	.14*	.05	.13*	.20**	1
Possible Scale Range	1–16	0–13	1–7	1–5	1–5	1–5	1–5	1–5	1–5	1–5	1–9	1–5
Mean	12.04	6.65	1.99	4.46	4.11	4.56	4.66	4.46	4.39	4.38	7.72	4.62
Standard Deviation	2.13	2.95	0.65	0.45	0.58	0.48	0.35	0.62	0.45	0.60	0.35	0.56
Observed Scale Range	1–16	0–13	1–4	2–5	2.3–5	3–5	3.3–5	1.7–5	2.8–5	2.5–5	6.4–8.5	2–5

Spearman’s rho correlations. N = 318 (except as follows: ^a^N = 314; ^b^N = 308; missing data was due to the option to select “unable to evaluate”). PDM = Professional Decision-Making in Medicine.

*p < .05;

**p < .01.

Form A scores were not associated with social desirability (r = .03, p = .954). Greater moral disengagement was associated with lower PDM scores (r = -.16, p < .01). Scores were modestly associated with higher PSPQ honor and integrity scores (r = .11, p = .060), but not significantly; the remaining associations with PSPQ scales were quite weak. PDM scores correlated positively with the peer interactions rating (r = .13, p < .05), but were not significantly associated with the clinical clerkships rating (r = .08, p = .168).

Given three very low scores (i.e., scores of 4, 2 and 1) in the PDM distribution, we examined the sensitivity of the correlations without those scores. The correlations with demographic variables and social desirability were virtually unchanged (N = 315). The pattern of correlations with moral disengagement (r = -.14, p < .05), honor and integrity (r = .09, p = .117), and peer interactions (r = .12, p < .05) remained consistent, but weakened. We think it is appropriate to retain low scores on the PDM, as identifying low scoring respondents is one purpose of a mastery test.

## Discussion

Our aim was to develop a content valid professional decision-making in medicine measure (Form A) and provide preliminary evidence for its construct validity. We also developed a second, alternate measure (Form B) and collected evidence of its equivalence to Form A. These new measures could be used in educational assessment in a pre-post design, and they could also be used for research on professional decision-making in medicine. In research, either Form A or Form B could be utilized to assess professional decision-making, so as to not overly burden research participants.

Our approach to assessing professionalism is unique because it examines decision-making strategies that professionals can apply across challenging situations. This approach is consistent with three key professionalism frameworks—virtue-based, behavior-based, and professional identity formation—currently reflected in medical education [58]. Equipping physicians with strategies for professional decision-making should support them in adhering to values and engaging in ethical reasoning during challenging situations [10]. In turn, this should help them engage in appropriate actions, as ethical decisions are precursors to ethical action [36]. Finally, collectively socializing medical students to employ decision-making strategies would foster an awareness of the necessity of such strategies as a physician during identify formation [58].

To ensure the content and face validity of the PDM, we developed items that mapped to a comprehensive framework of domains of medical professionalism and ethics that arose from a literature review and was verified through physician interviews [59]. Several relationships are suggestive of construct validity. PDM scores did not correlate with social desirability. Individuals scoring higher in moral disengagement scored lower on the PDM. PDM scores correlated only modestly with the PSPQ honor and integrity scale [51]. The remaining near zero correlations of PSPQ scales with PDM scores suggests the PDM measures something distinct from attitudes. However, both attitudes and decision-making strategies may be pathways that influence professionalism. Physicians must appreciate principles like respect and accountability, and they must also demonstrate skills and behaviors necessary for professionalism [51, 60, 61]. That is, attitudes might foster the motivation to behave professionally, but engaging decision-making strategies offers a means to demonstrate professional behavior in response to challenging situations.

Range restriction likely attenuated correlations with professionalism ratings, especially the clerkships rating. Additionally, professionalism in the clerkship rotations was operationalized as “reliability, initiative, honesty, integrity, and punctuality,” which may explain the lack of associations, as such behaviors might be more related to constructs like personality (e.g., conscientiousness) than to use of decision-making strategies. However, PDM scores were modestly correlated positively with the peer interactions professionalism rating. Indeed, the peer interactions rating had the greatest variance of the two professionalism ratings. This association suggests scores on the PDM may be predictive of behavior, but more data is needed. Evidence that professionalism assessment scores relate to subsequent clinical behavior helps to validate the importance of educational programs [61].

Overall, we found that the fourth-year medical students, on average, answered 75% of items correctly on a new professional decision-making measure. This reflects adequate performance. However, the PDM is a mastery-type test, and scores of at least 80%, ideally 90% or greater, would reflect much greater proficiency. In this study, moral disengagement demonstrated the largest association with professional decision-making scores. However, the relationship was modest. Thus, future research should examine additional factors that might enhance, or undermine, professional decision-making in medicine.

Quantitative research is well-suited to explore individual differences in variables such as knowledge, attitudes, values, and personality, in addition to educational or workplace experiences, such as exposure to unprofessional behavior [37]. The PDM focuses on cognitive strategies in social situations. Thus, social-cognitive variables such as cognitive flexibility, self-awareness, empathy, and perspective-taking may be fruitful avenues for research aimed at understanding responses to these situations [62–66]. We also recommend research examining PDM scores of physicians at different career stages and in different specialties. This research could be cross-sectional examining scores among physicians at different career levels. However, it would also be of value to explore longitudinally how medical students’ scores change over time with real-world experience in medicine. These differences could be examined in different specialties and medical practice settings (e.g., community medicine versus academic medicine).

Future research might also employ qualitative methods to follow up on these findings. This research might invite respondents to explain their rationale for particular choices, and also articulate their understanding of the issues at play within the vignettes. This research could identify whether medical trainees vary in recognizing the problems within the situations, and if they vary in how appropriate they view different decision strategies. For instance, a qualitative interview might uncover why particular strategies, like seek help or manage emotions, are viewed as less desirable. If cultural backgrounds do inform thinking about these situations, qualitative methods would be well-suited to identify these differences. For instance, some strategies, like seeking help, may vary in their cultural acceptability [67].

Regardless of individual characteristics that inform professional decision-making, educational research aimed at fostering decision-making strategies is of value. Individual characteristics may not be highly malleable, while teaching individuals no matter their career stage to adopt specific strategies when they encounter professional challenges is feasible [32]. This research would examine scores on the PDM Form A prior to an educational program, and examine if scores on Form B change after the program. For individual learners, strategy preference scores and the overall PDM score could be used for formative purposes to provide students feedback [61, 68]. The PDM is relevant to numerous contexts, whether learners are early in their careers or well-established professionals.

Future research should also address limitations of the current study. Specifically, we recommend additional data from respondents on both forms to establish them as parallel. The two forms’ mean scores were not statistically different and the parallel form correlation of .61 provides some initial support for their equivalence. Additionally, behavioral professionalism ratings focused directly on individuals’ decision-making by the same (and multiple) raters within the same clinical setting would provide stronger evidence of criterion-related validity. We used professionalism ratings data available to us in this initial study.

## Conclusions

Fourth-year medical students scored adequately on a new measure of professional decision-making that reflects strategies that compensate for constraints like bias, negative emotions, and lack of information. However, mastery of these strategies would be reflected in near-perfect scores. Of the strategies measured by the PDM, the participants tended to select “seek help” and “manage emotions” strategies much less than the others. Overall, routinely applying this set of strategies should assist professionals at any career stage with handling complicated professional challenges. The PDM is useful for studying the causes, correlates, and outcomes of professional decision-making in medicine. Future research should examine PDM scores, and obtain additional validation evidence, among physicians at different career stages. The PDM instrument has two test forms, making it suitable for educational assessment of programs that aim to teach professional skills like bias management and perspective taking. Clear delineation of what is to be measured is essential when assessing and studying medical professionalism. We hope that this study advances the discourse by presenting medical educators and researchers with a new framework for professional decision-making and an accompanying instrument.

## Supporting information

S1 File PDM data file. This file is an SPSS data file containing the anonymized dataset of 318 PDM Form A scores and 63 PDM Form B scores, along with item-level data for the PDM Form A and B, and data from the demographic questionnaire and other measures collected to examine the validity of the PDM measure.
(SAV)

S1 Fig PDM Form A distribution of scores. Illustration of the distribution of PDM Form A scores, which are negatively skewed.
(PDF)
