# Supplementary figures and images for: Professional decision-making in medicine: Development of a new measure and preliminary evidence of validity
Source: PLoS One. 2020 Feb 7;15(2):e0228450. doi: 10.1371/journal.pone.0228450 (PMC7006897; doi:10.1371/journal.pone.0228450)

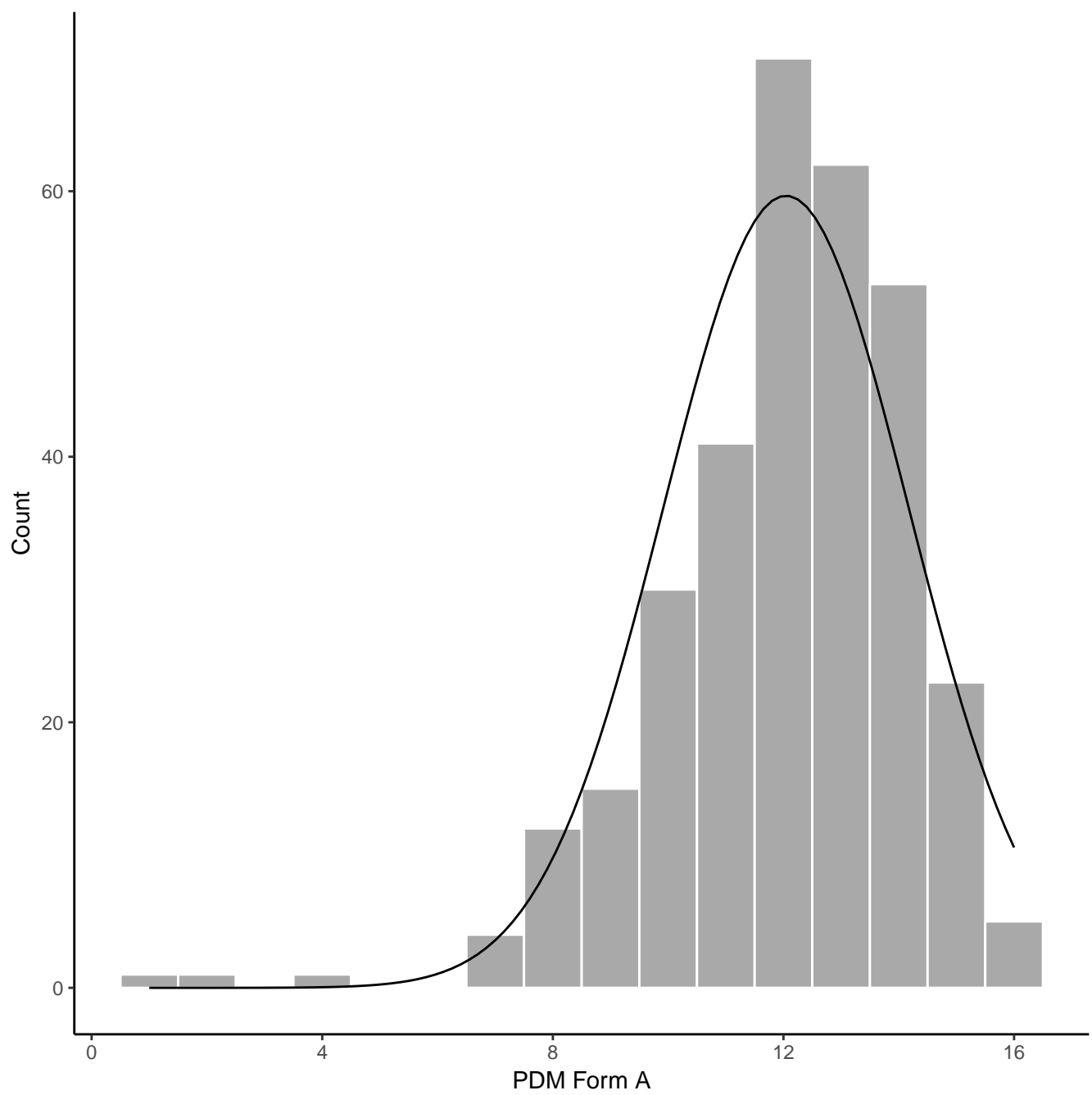

Supplement: S1 Fig — Illustration of the distribution of PDM Form A scores, which are negatively skewed. (PDF) [file pone.0228450.s002.pdf]
